# Computational Approaches of Quasi-Static Compression Loading of SS316L Lattice Structures Made by Selective Laser Melting

**DOI:** 10.3390/ma14092462

**Published:** 2021-05-10

**Authors:** Ondřej Červinek, Benjamin Werner, Daniel Koutný, Ondřej Vaverka, Libor Pantělejev, David Paloušek

**Affiliations:** 1 Institute of Machine and Industrial Design, Brno University of Technology, Technická 2896/2, 616 69 Brno, Czech Republic; Daniel.Koutny@vut.cz (D.K.); Ondrej.Vaverka@vut.cz (O.V.); palousek@fme.vutbr.cz (D.P.); 2 Institute of Lightweight Design and Structural Biomechanics, Vienna University of Technology, Gumpendofer Straße 7, 1060 Vienna, Austria; bwerner@ilsb.tuwien.ac.at; 3 Institute of Materials Science and Engineering, Brno University of Technology, Technická 2896/2, 616 69 Brno, Czech Republic; pantelejev@fme.vutbr.cz

**Keywords:** selective laser melting, finite element analysis, body centered cubic, quasi-static compression test, stainless steel 316L

## Abstract

Additive manufacturing methods (AM) allow the production of complex-shaped lattice structures from a wide range of materials with enhanced mechanical properties, e.g., high strength to relative density ratio. These structures can be modified for various applications considering a transfer of a specific load or to absorb a precise amount of energy with the required deformation pattern. However, the structure design requires knowledge of the relationship between nonlinear material properties and lattice structure geometrical imperfections affected by manufacturing process parameters. A detailed analytical and numerical computational investigation must be done to better understand the behavior of lattice structures under mechanical loading. Different computational methods lead to different levels of result accuracy and reveal various deformational features. Therefore, this study focuses on a comparison of computational approaches using a quasi-static compression experiment of body-centered cubic (BCC) lattice structure manufactured of stainless steel 316L by selective laser melting technology. Models of geometry in numerical simulations are supplemented with geometrical imperfections that occur on the lattice structure’s surface during the manufacturing process. They are related to the change of lattice struts cross-section size and actual shape. Results of the models supplemented with geometrical imperfections improved the accuracy of the calculations compared to the nominal geometry.

## 1. Introduction

### 1.1. Lightweight Structures

Lightweight structures and materials have become interesting for industries including transportation, aerospace, and space applications [1]. One category of lightweight structures are metallic cellular structures where metal foams, honeycombs, and lattices belong. Properties like low thermal conductivity, acoustic absorption, mechanical vibration damping, high stiffness to volume fraction ratio, and energy absorption are required within these materials [2,3,4]. Some of these properties are well represented by closed-cell and open-cell metallic foams [5]. However, both topological configurations are mostly irregular, which can cause randomly distributed damage during loading [6]. Furthermore, closed-cell foams form gas capsules that are usually non-desirable, and open-cell foams tend to be deformed by bending instead of the more convenient stretching-dominated mode [7,8,9].

Additive manufacturing (AM) brings new production possibilities of stretching-dominated lattice structures with enhanced mechanical properties such as high energy absorption related to their weight or damping properties [10,11,12]. One of the frequently used AM technologies called selective laser melting (SLM) allows manufacturing from different materials, e.g., stainless steel 316L [13], titanium alloy Ti_6_Al_4_V [14], or aluminum alloy AlSi_10_Mg [15,16].

With the AM, the topology of lattice structures can be customized to a wide range of applications, including a different kind or direction of loading behavior [17,18]. This advantage can be used when lightweight components are designed for the transmission of accurately defined loading [8,19]. Structures can also be designed to absorb a specific amount of energy and undergo predefined deformation patterns [20]. A deeper knowledge of loading behavior and manufacturing technology is required to increase the efficiency of lattice structures, for example, in terms of energy-absorbing capabilities [21]. It includes information about the deformation mechanism within a specific geometry configuration, which can be investigated via finite element analysis (FEA) [22]. Significant inaccuracies and imperfections can occur [23], as far as the geometry of lattice structures produced by SLM technology is strongly influenced by the heat transfer phenomenon. In the computational model of lattice, the structure geometry should be considered with deviations from the nominal computer-aided designed (CAD) data [24].

### 1.2. Computational Approaches

Some methods of finite element discretization of geometry have been developed to study the properties of lattice structures under mechanical loading. Luxner et al. [25] focused on the uniaxial compression properties of lattice structures with circular cross-sections and constant diameter. Timoshenko beam element models were utilized for the simulation of large structures. A stiffness correction in the vicinity of the vertices was introduced by using elements with artificially increased Young’s modulus in these domains [26,27]. For a highly detailed representation of the structure topology, solid tetrahedron elements were used, giving higher modeling effort and computational cost.

Ravari et al. [28] developed a Python 6.6.6. script for creating models of lattice structure geometry using quadratic beam element B32 and solid tetrahedron elements C3D10M from the Abaqus library. The script divided the lattice struts into at least 9 equally spaced intervals variating in the strut’s diameter with a circular cross-section. A diameter according to probability was assigned to each interval. Furthermore, Dong et al. [29] dealt with the concept of loading only a single strut in the structure. To indicate the influence of the joint on the stiffness of the lattice, strut, beam, and solid element models were generated. A similar approach was invented by Geng et al. [30] who used finite element models based on combined elements. Some of the Timoshenko beam elements in a unit cell in the middle of the loaded lattice structure were replaced with solid tetrahedral elements C3D10.

Vrana et al. [31] described methods of optical digitalization to achieve a model of lattice structure with the actual manufactured cross-section area and shape. The actual shape of the tilted lattice struts produced by SLM was approximated by an ellipse. Besides, Lei et al. [24] used micro computed tomography (μ-CT) to capture the realistic geometrical information of the lattice strut surface including imperfections. A Python script was developed to automatically create a 3D model using B31 beam elements. Actual distribution characteristics of imperfection were taken into account in the FEA model as the opposite of Ravari et al. [28]. The quasi-static FE simulations were conducted in the ABAQUS 6.14 Explicit solver. The same solver used also Gümrük et al. [32], Li et al. [33], and Liu et al. [34] for solving quasi-static compression behavior of body-centered cubic (BCC) lattice structure under large deformation. The ratio of artificial energy to internal energy and the ratio of kinetic energy to internal energy were held below 5% to ensure that the dynamic effect is insignificant. The different models presented by Lozanovski et al. [35] captured the ‘waviness’ of a struts’ varying diameter along its length. They used a series of elliptical cross-sections derived from µ-CT measurements to develop a model geometry that includes manufacturing imperfections.

Several teams focused also on the development of a model of the material. Tsopanos et al. [36] used tensile tests of thin struts complemented with compression tests of BCC lattice cubes. The experimental results were used to obtain the mechanical properties of the structure produced by SLM. The elastic modulus varied until a good match between the finite element analysis and experiment was achieved. This knowledge used Smith et al. [3] for numerical modeling of the lattice structure compressive response. Initially, the material properties based on conventional tensile tests and a strut diameter of 0.2 mm was used for the FE models. The strut diameter then varied until both the experimental and FE stress-strain curves coincided. The ends of the struts were modeled with an increased diameter similar to the Luxner et al. [25] study.

Tancogne et al. [10] used a piece-wise linear hardening curve (a simple rate-independent J2-plasticity model with isotropic hardening) based on calibrated tensile experiments performed on SLM-made samples for a description of lattice structures made of SS316L. The effect of possible anisotropy, rate dependency, kinematic hardening, and martensitic phase transformation was neglected. A similar approach was used by Gümrük et al. [32]. Tancogne et al. [19] continued the research with the numerical investigation of the BCC lattice structure with tapered beams. Simulations were performed on a single unit cell to investigate the effect of tapering on the elastic moduli and the structure when large deformation occurs. The base material was a homogeneous isotropic Levy-von Mises material with isotropic strain hardening.

The material model was improved by Amani et al. [37], who studied the compression behavior of face-centered cubic (FCC) lattice structures manufactured by SLM. The deformation process of the lattice was captured by in situ and ex situ X-ray tomography illustrating a macroscopic structure and local micro-porosity. A 3D image-based conformal finite element model was then built for the simulation of the compression test using Gurson–Tvergaard–Needleman (GTN, in Abaqus) porous plasticity (based on von Mises yield criterion of ductile porous materials). A new procedure allowing to inform each element about the local porosity directly from high-resolution tomography was used. Simulation considering a homogenous matrix with an average initial porosity everywhere was compared to the new heterogeneous model.

Unfortunately, published studies usually involve simulations considering of only some of the most important characteristics of lattice structures. Some authors focus on the correct determination of input data for the model of the material using numerical corrections usually restricted to linear elastic behavior. Other researchers focus on the exact determination of the model of geometry including imperfections using µ-CT. These studies usually compare the mechanical properties of lattice structures in terms of compression modulus or collapse stress. However, a study with correctly determined models of material and geometry beyond the linear elastic area, is missing. The description of lattice structure behavior in the nonlinear area is crucial for future applications that consider progressive collapse, e.g., energy absorbers in the transport industry. Therefore, the main objective of further research should aim to the development and verification of computational models that allow the prediction of lattice structures’ nonlinear deformation. The models will allow the designing of vehicle protection segments using lattice structures with minimal experimental effort. This study focuses on the development of computational analysis considering the abovementioned input parameters and the determination of their relevancy compared to experimental data.

## 2. Materials and Methods

**Figure 1:**
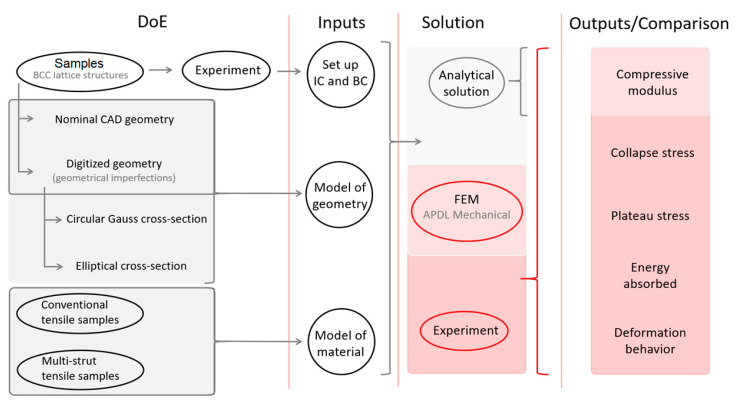
Scheme of the main working points in study.

To realize the aim of this study, it is necessary to perform a series of procedures connected to material testing, optical digitalization, and finite element analysis. The main processes described in the following sections are shown in the scheme in Figure 1.

### 2.1. Powder Material

For the production of lattice cubes and multi-strut tensile samples, the 1.4404 (316L) stainless steel metal powder (TLS Technik GmbH, Bitterfeld-Wolfen, Germany) was selected. The manufacturability of this material has reached a level allowing the production of parts with complex geometry such as lattice structures. Furthermore, stainless steel 316L is a ductile material with high elongation at failure (41 ± 1% without heat treatment [38]), which predetermines it to a good resistance during loading assumed for energy absorption. The chemical powder chemical composition of the 316L is given in Table 1. The gas atomization method was used to produce the powder. The particle size requirement was given by values between 15 µm and 45 µm for 50 µm layer thickness. The powder particle size analysis showed a distribution with the following characteristics that met the expectations: *Q*_10_ = 10.07 µm, *Q*_50_ = 29.44 µm, and *Q*_90_ = 48.21 µm.

### 2.2. Lattice Structure

**Figure 2:**
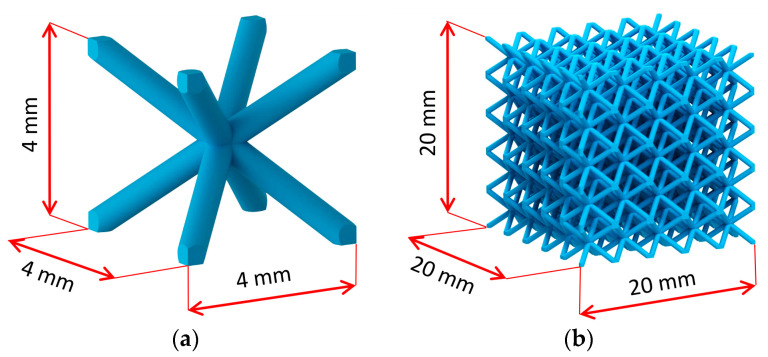
(**a**) BCC unit cell; (**b**) BCC lattice structure sample.

The present study focuses on a basic lattice structure that has a unit cell assembled by four struts along the body diagonals of a cube (see Figure 2a), which is typically called body-centered cubic (BCC). Lattice structured cubes with nominal dimensions of 20 × 20 × 20 mm and 4 mm unit cell size (see Figure 2b) were designed for a quasi-static compression test. The nominal strut diameter of the structure in CAD design varied from 0.3 mm to 1 mm. The samples were manufactured using an SLM 280^HL^ machine (SLM Solutions, Lübeck, Germany) with the following standard set-up process parameters according to SLM Solutions recommendations: 100 °C platform heating, N_2_ inert atmosphere, bidirectional hatching scanning strategy with two contours, and 50 µm layer thickness. The melting parameters were: 100 W laser power and 300 mm·s^−1^ scanning speed for scanning contours, 275 W laser power and 700 mm·s^−1^ scanning speed for hatching, 150 W laser power, and 400 mm·s^−1^ scanning speed for fill contours.

### 2.3. Multi-Strut Tensile Samples

**Figure 3:**
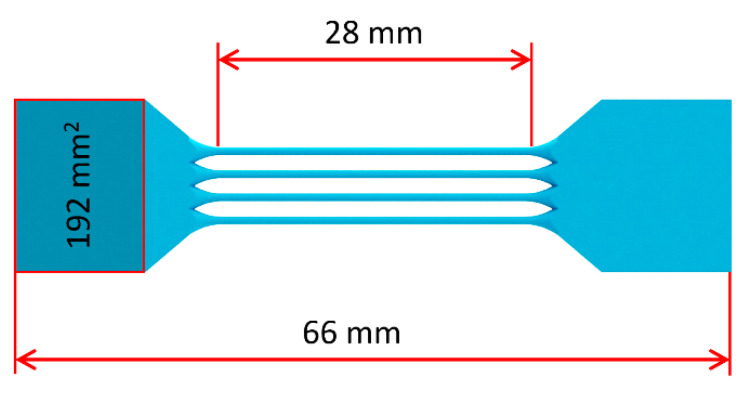
Multi-strut tensile sample with 12 struts.

Tensile tests were carried out to determine the mechanical properties of 316L stainless steel. Conventional tensile samples manufactured according to usually used Standards (ISO, DIN) are not representing the mechanical properties of the lattice structure closely enough [36,38]. Already mentioned in Vrana et al. [39], the surface area of all parts manufactured via SLM technology is influenced by heat transfer and other phenomena during the manufacturing process. Therefore, this area is characterized by different values of mechanical properties. The percentage portion of these areas in samples manufactured according to abovementioned standards is significantly lower compared to the lattice structure struts. Therefore, strut tensile samples with a nominal strut diameter equal to the lattice structure struts were used (see Figure 3).

Furthermore, the manufacturing angle of the samples was considered. According to Koutny et al. [40], the actual strut diameter measured after the manufacturing process differs from the nominal ones. Actual shape and size depend on many aspects, e.g., the settings of process parameters, powder distribution, manufacturing conditions, and manufacturing angle [23]. All tensile samples were manufactured with a 35° angle regarding the platform to achieve similar strut manufacturing conditions as in the BCC lattice structure. This ensures (together with equal process parameters) a very similar strut diameter, shape of cross-section, and mechanical properties of the multi-strut tensile samples compared to the BCC lattice structures.

Besides the multi-strut tensile samples, a series of conventional samples was manufactured with the same process parameters. Testing samples were prepared from billets built with a 90° angle regarding the platform and machined according to DIN 50125—(Form B, dimensions of the gauge length Ø6 × 30 mm).

### 2.4. Dimension and Shape Analysis

The previous series of structured samples was used to inspect the actual diameter and cross-section area of the struts after the manufacturing process. After post-processing, these samples were subjected to the optical digitalization process. An optical scanner ATOS Triple Scan (GOM GmbH, Braunschweig, Germany) with an MV170 lens was used (calibration was carried out according to VDI/VDE 2634). The samples were coated with titanium dioxide powder before scanning to prevent reflection of light projection (coating thickness approx. 5 µm [41]). The samples were scanned on the rotation table using a script written for maximizing the total area of the scanned surface.

**Figure 4:**
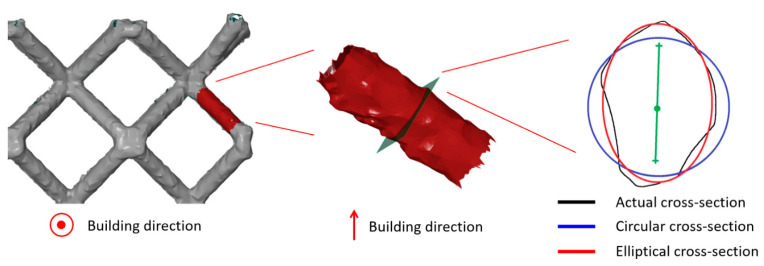
Comparison of different cross-section approximations.

The scans of lattice structures were evaluated using GOM Inspect v8.0 software. Eight measurements were carried out on struts at middle height (corner struts) for every sample. These struts were interlaced with cylinders based on the Gaussian best fit method (points 3 sigma) [40]. Diameters of the cylinders were measured. In the next step, the struts were cut at half of their length. The cross-section created by the section plane was interlaced by an ellipse with the same Gaussian best fit function (see Figure 4), and the major and minor axis diameter of the ellipse were determined.

### 2.5. Quasi-Static Mechanical Tests

Tensile tests (multi-strut samples, standard tensile samples) and compression tests of lattice cubes were performed on a universal testing machine Zwick Z250 (ZwickRoell GmbH & Co. KG, Ulm, Germany) equipped with dynamometer enabling load of 150 kN. The declared positioning accuracy of the device measurement (with repeatability) is ±2 µm. Ends of multi-strut tensile samples were fixed into the centered jaws and preloaded with 20 N force. Self-locking grips prevented the slipping of samples during the test.

The lattice cubes were placed without any fixing between the flat adapters in the device. The lower adapter was fixed on a bar movable in a vertical direction and the upper adapter was mounted on a static joint connection (slight tilting of the adapter was allowed). Both tensile and compression samples were loaded with a strain rate of approximately 10^−3^ s^−1^; therefore, no strain rate effect was expected.

### 2.6. Analytical Formulation

Analytical approaches were developed for the simplified evaluation of cellular structure deformation behavior [2,42]. This study is using one of the newest approaches presented by Yang et al. [43], which accounts for the unit cell length, nominal strut radius, and boundary conditions of the BCC lattice structure. Equations described in this study were based on the earlier Timoshenko beam theory and Euler–Bernoulli theory [19] neglecting shear deflection terms. The equations used in this study do not contain the boundary plates constraints of the investigated lattice structure representing free strut deformation patterns (Equations (1) and (2)). With these equations, an elastic modulus $E_{c1}^{e}$ of lattice structure is calculated as follows: 

Timoshenko solution

(1) $$E_{c1}^{e}=\frac{9\sqrt{3}\pi E_{s}}{\left(17+12\upsilon_{s}\right){\left(\frac{l}{r_{n}}\right)}^{2}+2{\left(\frac{l}{r_{n}}\right)}^{4}}$$

Euler–Bernoulli solution

(2) $$E_{c1}^{e}=\frac{9\sqrt{3}\pi E_{s}}{3{\left(\frac{l}{r_{n}}\right)}^{2}+2{\left(\frac{l}{r_{n}}\right)}^{4}}$$

where $E_{s}$ is the elastic modulus of bulk material, $\upsilon_{s}$ is Poisson’s constant of bulk material, *r_n_* is strut radius, and l is half of the unit cell diagonal. It should be mentioned that the analytical models take into account only nominal strut radius; therefore, the imperfections connected with change of cross-section area and its shape are neglected here.

### 2.7. Finite Element Analysis

Numerical simulations were carried out in ANSYS Workbench 19.2 in module for structural analysis (Static structural). The subject of the simulation was a quasi-static compression test of the BCC lattice structure produced by SLM technology. Two different approaches were introduced and compared with experiments, including linear and nonlinear deformation behavior. The beam element model was developed as a computationally cheap solution for the simulation of bigger structures. For analysis requiring higher accuracy and stress analysis, a solid element model was used. Manufacturing imperfections connected with the change of strut cross-section and cross-sections’ shape were considered in the beam element model simulation.

#### 2.7.1. Solid Element Model (Continuum Model)

The model of geometry consisted of tetrahedron elements (SOLID 187) with a quadratic interpolation function. This approach is computationally expensive and is restricted to smaller bodies, but gives information about the stress evolution in the lattice structure during loading. Because of higher computational requirements, a mesh sensitivity study was performed on a smaller lattice structure with a configuration of 3 × 3 × 3 unit cells to achieve reasonably accurate results [35]. During the study, the level of plateau stress and the convergence of the solution were validated through the series with different element sizes [3,37,43]. At least six elements were used to discretize the diameter of the strut for geometry with and without imperfections [19,26]. Struts in the model with imperfections were represented by a constant elliptical cross-section based on measurement. Nodes created by intersecting struts were modelled with sharp corners without radiuses.

#### 2.7.2. Beam Element Model

**Figure 5:**
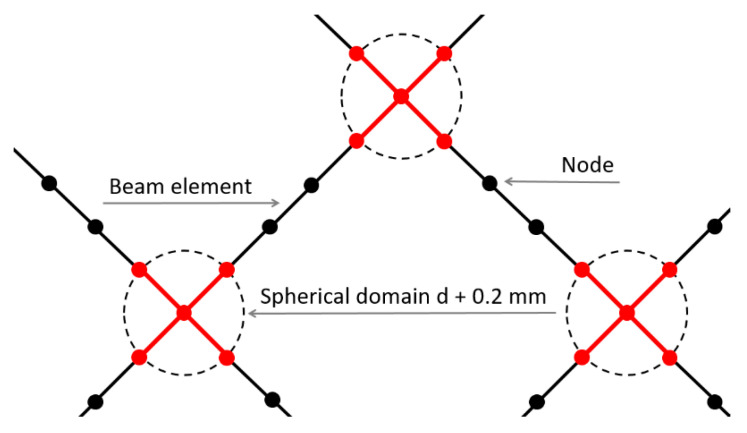
Schematic composition of elements and nodes with different properties.

**Figure 6:**
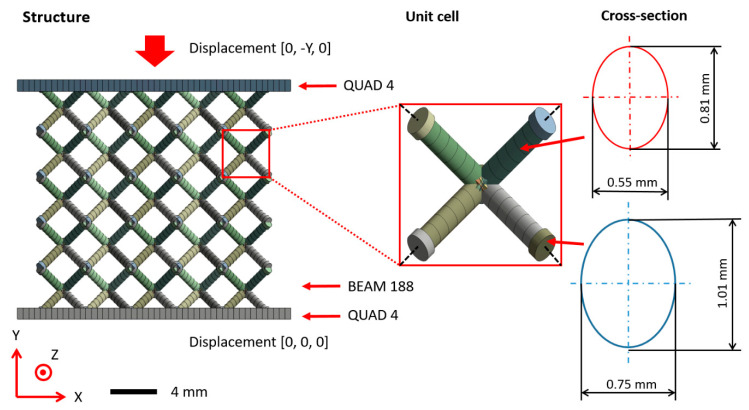
Segmented beam element model of BCC lattice structure including geometrical imperfection (for 0.6 mm nominal strut diameter).

The model was created using a script written in APDL by copying a single unit cell represented by a wireframe. Struts of unit cells were divided into mid-part and ends. Each strut consisted of minimal five beam elements (BEAM 188) according to the mesh sensitivity study performed in previous studies [30,44]. The behavior of nodes was adjusted at nodes where at least three ends of the struts met [26]. This step was done to achieve more realistic behavior of intersecting struts represented by the spherical domain rather than one point. It is caused by additional material accumulated in the struts after the manufacturing process. It included higher stiffness and material volume increase in the near vicinity of nodes. The artificial increase of stiffness was achieved by a thousand times increased value of Young’s modulus. The higher material volume was achieved by an increase in nominal strut diameter about 0.2 mm (see Figure 5 (red)). This approach ensures bending and cracking struts rather than reinforced nodes during compression loading [45,46]. This approach is based on previous studies such as Luxner [26], Labeas [47], Smith [3], and Gümrük [32], and supplemented with imperfections of the manufacturing process (see Figure 6).

Besides the lattice structure, the FE model included a top and bottom surface discretized with quadrilateral shell elements (SHELL 181), where boundary conditions were applied [24]. A standard Structural Steel model was assigned to the shells supplemented with a thousand times higher values of Young’s modulus to account for the rigidity of adapters [26]. Between the beam elements of the lattice structure and the top and bottom surfaces, contact with a static friction coefficient [10,35] of 0.15 was applied (tabular steel-steel contact).

The model of the material of the lattice structure was based on a quasi-static tensile test of multi-strut samples. The data obtained by tensile test in the form of force-displacement curves were further evaluated. The optical scanning methods described in Section 2.4 were used for the determination of the actual cross-section of struts in the multi-strut sample. Results were used for the construction of a simple nonlinear elastic-plastic model of the material with isotropic hardening according to stress-strain curves [24,25,37]. No failure criterion was considered due to the ductile properties of stainless steel 316L, which preserved the continuity of structure, even under large deflection [18].

The compressive loading was applied as a displacement in the y-direction on the top surface. In addition, the bottom surface was constrained in all degrees of freedom. Except for this movement, no other constraints were applied. Quarter-symmetry conditions cannot be introduced to make possible a small sliding structure along the diagonal during its deformation. The step end time was set to 1 s during one step and auto time stepping to program control. Large deflection settings were turned off.

## 3. Results and Discussion

### 3.1. Strut Dimension Analysis/Samples Morphology

**Figure 7:**
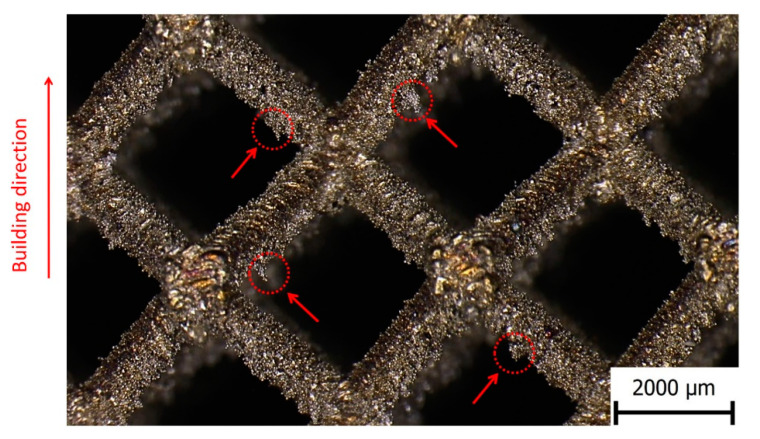
Detail of lattice structure surface with imperfections.

**Figure 8:**
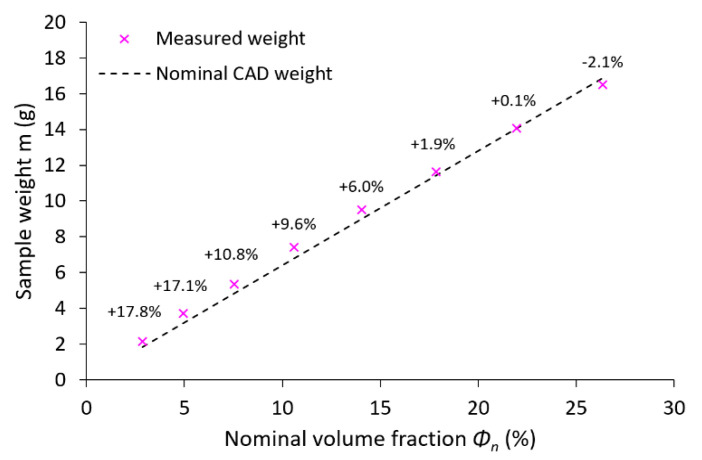
Comparison of the actual average and nominal weight of samples according to the nominal volume fraction; the numbers above points show the percentage of weight variation.

After-manufacturing weight inspection revealed variations in the mass of the samples compared to nominal weight values based on CAD data. A similar phenomenon was observed by Gümrük [32] within BCC lattice structures manufactured from the same material by SLM. This variation differed for all manufactured structures with a nominal diameter value between 0.3–1.0 mm. The residues of supporting cones used in the manufacturing process were excluded as a probable cause of the weight increment because support material was milled down during the post-processing phase to make the samples equally high. The detailed microscope photo shows a large number of metal particles melted on the surface of the sample strut. Most of these particles occurring on the bottom of the struts in the form of irregular clusters (see Figure 7, aggregates in red circles). It is caused by an increased heat transfer into the powder layer beneath, compared to the surrounding area. This phenomenon leads to a change of the geometry of down-skin surfaces known as the staircase (stair-step) effect [48]. The conditions of this effect led to a change of strut cross-section shape and size [3,21], dominantly in a direction perpendicular to the built platform. This led to a deviation in sample weight compared to nominal data (see Figure 8). It must be mentioned that the calculation of nominal weight is based on data not considering the strut porosity [48], which probably occurred during sample manufacturing. For standard process parameters tuned by the machine manufacturer, a negligibly low porosity value is assumed.

**Figure 9:**
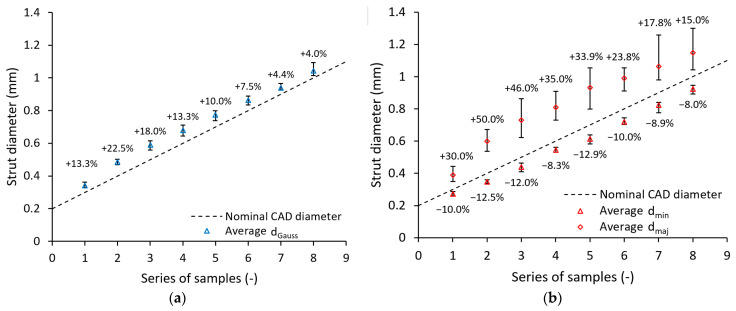
Difference between nominal and measured dimensions for (**a**) Gauss circular and (**b**) elliptical measurement; numbers above/beneath points show the percentage of dimension increase/decrease compared to CAD data.

The lattice structures were digitalized as described in Section 2.4 to determine the accurate strut cross-section geometry. Based on these measurements, the strut cross-section geometry was approximated with a circular (according to Gauss distribution) and an elliptical shape to represent the manufactured strut geometry more precisely. The diameter of both Gauss circular and elliptical cross-sections based on optical measurements is bigger than the nominal CAD diameter values for all samples. The increase of diameter for the Gauss circular approximation is thereby between +4.0% and +22.5% (see Figure 9a). The major axis of the elliptical cross-section varies between +15.0% and +50.0%. The minor axis for all strut sizes is slightly smaller than the nominal value (between −8.0% and −12.9%; see Figure 9b). These values are reflected in the load-bearing strut cross-section area (see Table 2). Together with the results of weight measurement and the knowledge from a previous study [41], the following can be concluded: there is probably a strut diameter beyond the range of diameters investigated in this study for which increments in cross-section area caused by imperfections became negligible if the trend remains. Detail assessment of the border nominal strut value for which geometrical imperfections connected to the change of its cross-section has to be considered and should be further investigated.

Optical measurements of struts in a previous study [40] revealed that an elliptical approximation is more accurate to the actual manufactured strut compared to a circular cross-section. Therefore, it was decided to use primarily the average values of elliptical measurements given in Figure 9b for creating models of geometry in numerical simulation. This approach allowed the introduction of one of the manufacturing imperfections with a crucial impact on lattice structure mechanical properties. According to the measurements, two geometrical configurations were adopted. The first considers only the change of circular strut diameter measured according to Gauss distribution (see Figure 9a), while the second also changes its shape to elliptical (see Figure 9b).

### 3.2. Multi-Strut Tensile Test Evaluation

**Figure 10:**
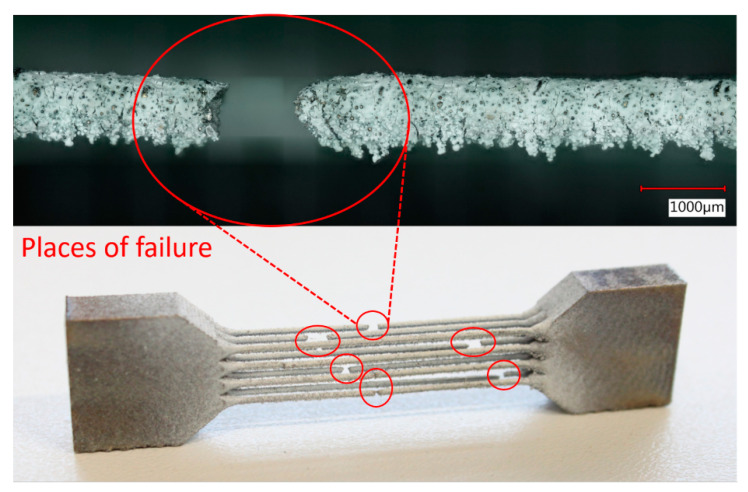
Multi-strut tensile sample after experiment including the detail of strut failure.

The engineering stress-strain curves as a result of the multi-strut tensile tests are related to the sum of all strut cross-sections in the sample. Strut dimensions and their actual cross-sections were measured by optical digitalization methods similar to those used for the lattice structure struts measurement. The samples were loaded until all 12 struts in the sample were broken (see Figure 10). All struts failed in different heights of the sample, which indicates approximately homogeneous mechanical properties across its length. This phenomenon is in contrast to conventional samples, which usually fail in the diagonal direction. The failures were probably driven by the random distribution of larger pores in thin struts, which caused local weakening of cross-sections. On the other hand, if the failure manners of separate struts are judged, a trend similar to the conventional samples occurs.

Because the process parameters and the tilt angle of the multi-strut samples are identical compared to the struts in the BCC lattice structure, similar cross-section deviations, as well as mechanical properties, were expected. A comparison of the actual manufactured strut cross-section between the multi-strut tensile samples and the lattice structures revealed a deviation of the minor axis smaller than 25 µm. Based on the tensile tests of multi-strut tensile samples, true stress-strain values were calculated unencumbered by imperfections. From the calculated dependency, a bilinear elastic-plastic behavior was defined with Young’s modulus *E_s_* of 94 GPa, yield strength (0.2% proof stress) *R_p_*_0.2%_ of 338 MPa, and tangent modulus *E_t_* of 787 MPa (see Table 3).

Mechanical properties obtained by tensile tests of conventional and multi-strut tensile samples showed the following:

- Young’s modulus *E_s_* and yield strength *R_p_*_0.2%_ determined by testing of multi-strut tensile samples achieved only 57% and 75% of the conventional samples values;
- Elongation at failure *A* of multi-strut tensile samples was significantly lower compared to conventional samples, which is appointed to the increased fragility of the thin strut described in previous studies [3,32,36];
- Young’s modulus *E_s_* obtained by multi-strut tensile samples testing is approximately 49% lower compared to the results achieved by single strut samples testing combined with the numerical correction presented by Tsopanos [36] and Smith [3]. Contrary to this, yield strength *R_p_*_0.2%_ was more than two times higher compared to previous studies;
- A good correlation of mechanical properties between multi-strut samples test and Gümrük [32] study was found. Young’s modulus *E_s_* and yield strength *R_p_*_0.2%_ values deviated up to 5%;
- A good correlation of mechanical properties between conventional samples and the data sheet from SLM Solutions was found. Young’s modulus *E_s_*, yield strength *R_p_*_0.2%_, and elongation at break *A* values deviated up to 7%.

### 3.3. Comparison of Analytical Approaches and Experiment

**Figure 11:**
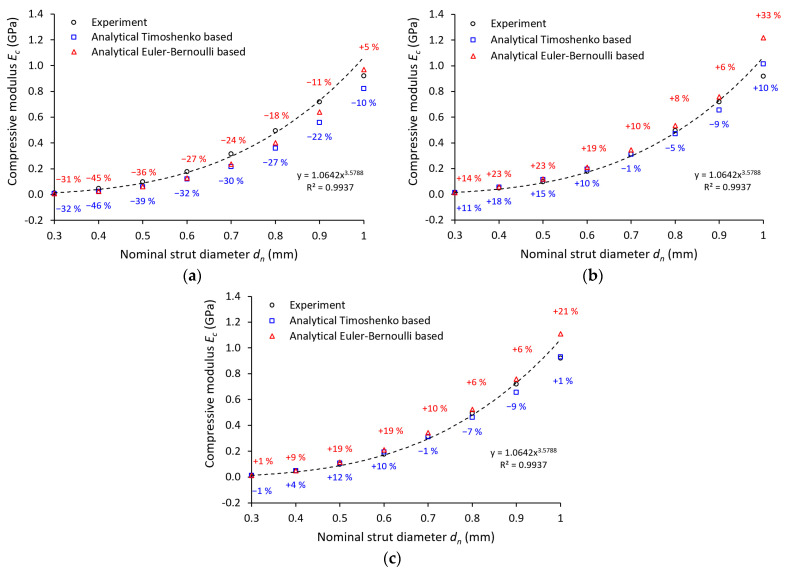
Comparison of quasi-static compression experiment with an analytical approach for: (**a**) nominal strut diameter; (**b**) Gauss circular approximation; (**c**) elliptical approximation.

For comparison of the experimental and analytical results, two different approaches considering the unconstrained movement of struts’ free ends introduced by Yunhui [44] were used (Equations (1) and (2)). To calculate the compression modulus of the lattice structure *E_c_,* Young’s modulus value *E_s_*, introduced in Table 3, with 94 GPa is used. Furthermore, the nominal strut cross-sections are applied without considering imperfections (see Figure 11a). The analytical approach based on the Euler–Bernoulli theory (see Equation (2)) predicts the results of *E_c_* closer to the experimentally determined compression modulus of the lattice. The analytical approach based on the Timoshenko beam theory (see Equation (1)) shows a similar trend but predicts a slightly smaller *E_c_*. For a nominal strut diameter of 0.4 mm, the results of both analytical approaches deviate the most compared to the experiment (45% and 46% lower).

This behavior can be attributed to the boundary conditions of the analytical approach. Both equations assume a frictionless contact on interfaces: the free ends of the lattice structure and the loading surface, and the free ends of the lattice structure and the supporting surface. On the contrary, in the experiments, the contact between the free ends and the surface of the testing machine adapter is characterized by contact with friction. Furthermore, the analytical model did not consider the imperfections of the manufacturing process. The only geometry involved in both models is the nominal strut diameter, which differs greatly from the actual ones according to abovementioned measurements (see Table 2). Therefore, two additional analytical computations were performed for Equations (1) and (2) considering the Gauss circular (see Figure 11b) and elliptical (see Figure 11c) strut cross-section. The input value *r_n_* for the elliptical cross-section was defined from the average major axis *d_maj_* and minor axis *d_min_* measurements.

The best agreement with the experiment was achieved by the analytical computations considering the elliptical cross-section for the Timoshenko equation. The biggest deviation from the average experimental values reached 12% at strut equal to 0.5 mm diameter. Contrary to this, the worst accuracy was determined by the computations considering the Gauss circular cross-section for the Euler–Bernoulli equation. The biggest deviation from the average experimental values reached 33% at a strut equal to 1.0 mm diameter. For a better assessment of the analytical approaches, further investigations in terms of structure morphology, boundary conditions, and geometrical imperfections must be done.

### 3.4. Comparison of FEM and Experiment

#### 3.4.1. Linear Material

**Figure 12:**
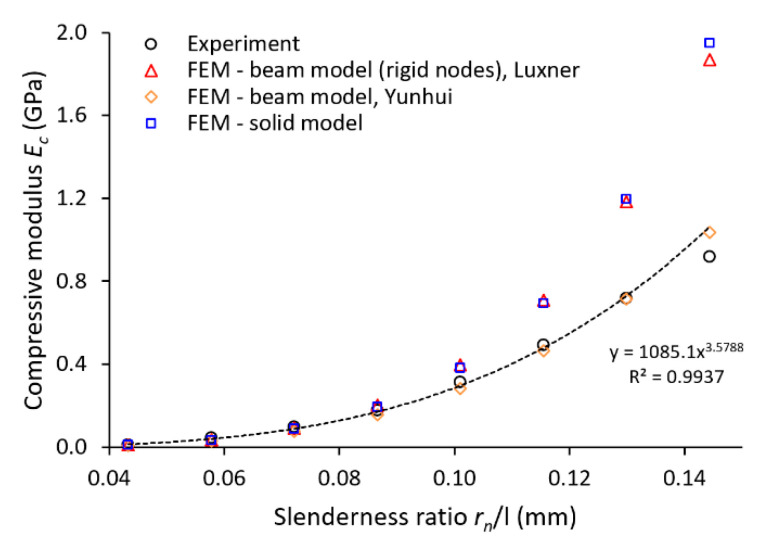
Comparison of lattice structure compressive modulus with a numerical solution.

**Figure 13:**
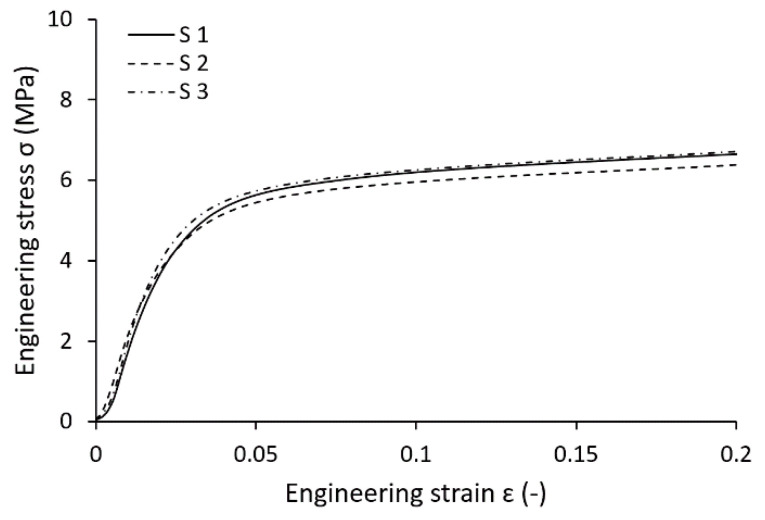
Compression test of BCC lattice cubes with nominal strut diameter 0.6 mm.

In the first step, the experiment was compared to FE analysis considering the nominal strut diameter with a circular cross-section. In addition, the material accumulation due to the manufacturing process was considered by increasing the strut diameter by 0.2 mm as described in Section 2.7. The model of the material was restricted to linear elastic behavior (Young’s modulus 94 GPa). The resulting structure compressive modulus (*E_c_*) versus slenderness ratio (*r_n_*/*l*) are shown in Figure 12, with the slenderness ratio defined as strut radius *r_n_* divided by the strut length *l*. As clearly visible from the results, the simulation is in good agreement with the experiment (FEM—Beam model, orange rhombus). An inaccuracy within the last value can be caused by experimental results deviating from the overall trend or lower stiffness of structures, which are then sensitive to deviations. Repeatability tests of mechanical properties showed a good correlation (range < 5% in plateau area, see Figure 13); nevertheless, better stability of the results can be achieved when more samples with the same nominal strut diameter are tested.

**Figure 14:**
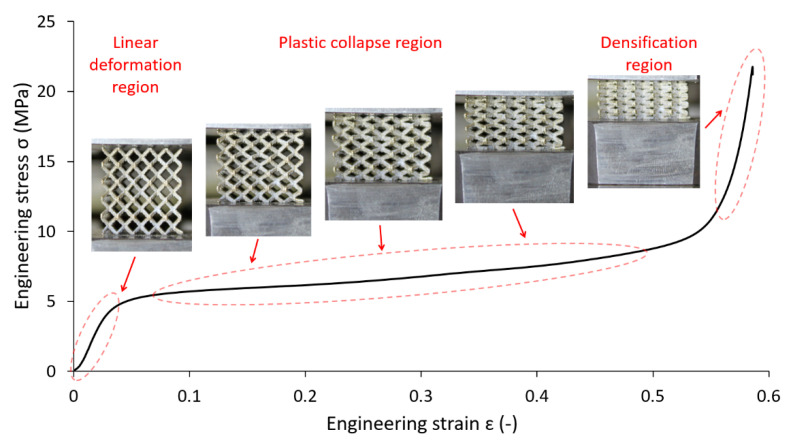
BCC lattice structure behavior in different regions of deformation.

This approach worked well in the linear deformation region (see Figure 14), and therefore, allowed us to compare the lattice structure properties in terms of compressive modulus or initial stiffness. On the other hand, it was not possible to inspect the internal stress evolution during the loading of the structure. Therefore, a solid element model was introduced (Figure 12, FEM—Solid model, blue squares) to simulate the realistic connection of struts in the vicinity of the node, which is rather represented by the domain than point connection. The loading and the boundary conditions remained the same. The numerical approach was, furthermore, supported with the beam element model (FEM—Beam model (rigid nodes), red triangles) with adapted stiffness (Young’s modulus ×1000) in the near area of nodes according to the Luxner study [26]. The length of the adapted node beams was equal to the nominal strut diameter increased by 0.2 mm (see Figure 5).

**Figure 15:**
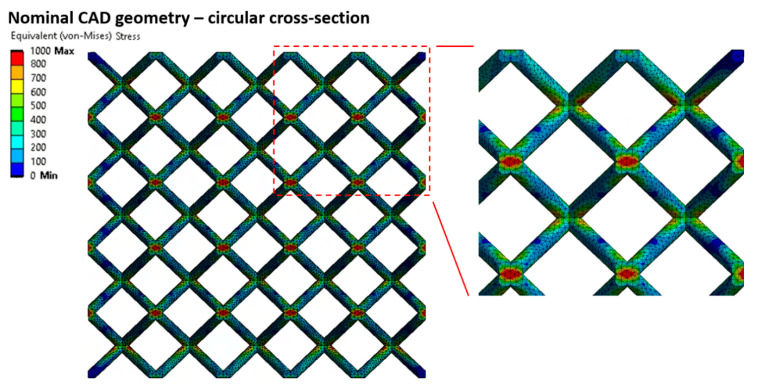
Equivalent (von Mises) stress distribution for a structure considering linear elastic material behavior—Circular cross-section (2.5% structure deflection).

**Figure 16:**
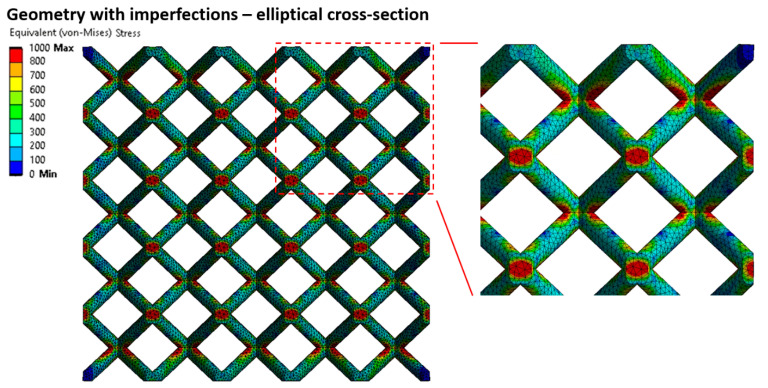
Equivalent (von Mises) stress distribution for a structure considering linear elastic material behavior—Elliptical cross-section (2.5% structure deflection).

The compressive modulus was calculated by both approaches with actual strut connection achieve similar values, but their difference compared to the experiment increased with the rising slenderness ratio. This difference is appointed to high stiffness when only the linear elastic behavior of the material is considered. The stiff behavior manifests, especially in the near vicinity of structure nodes, where the highest stress occurs during structure loading (see Figure 15 and Figure 16). On the other hand, it must be mentioned that the experimental values were determined at the beginning of the linear area of the stress-strain deformation curve with the assumption of nearly elastic linear material. However, the possible explanation could be that plastic deformations can also occur in this area. It could happen despite a linear manner because the area usually takes up to a few percent of the lattice deformation. Therefore, a comparison of the experiment has to be extended to the calculation of nonlinear elastic-plastic material behavior.

#### 3.4.2. Non-Linear Material

**Figure 17:**
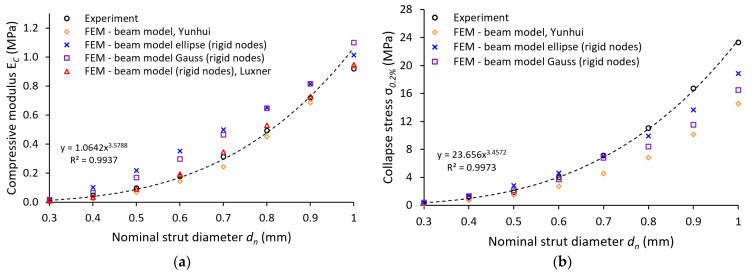

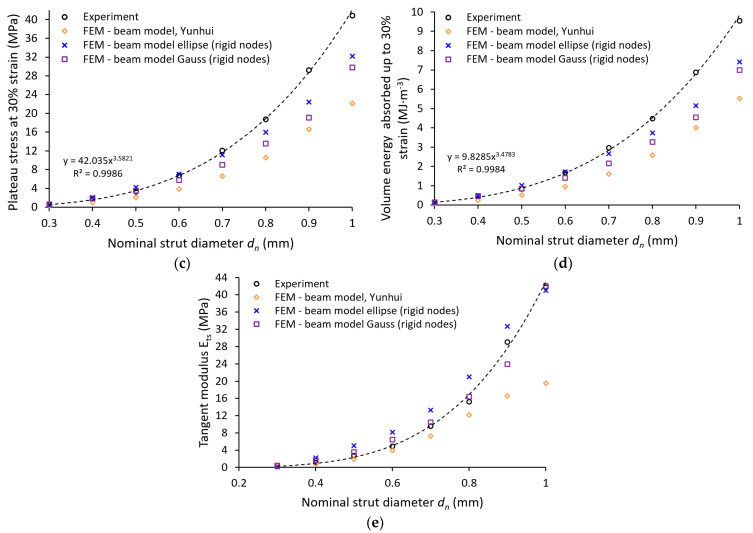
Comparison of quasi-static compression experiment with numerical solutions using different approaches—(**a**) compressive modulus; (**b**) collapse stress (0.2% structure strain); (**c**) Plateau stress; (**d**) volume energy absorbed; (**e**) tangent modulus of the structure.

Besides the beam element model with elastic-plastic material behavior (Figure 17 FEM–Beam model, orange rhombus), three additional beam element models with nonlinear behavior are compared to the experimental outcome. These include a model with rigid nodes and a nominal strut cross-section (FEM–Beam model (rigid nodes), red triangles), a model with rigid nodes and an elliptical strut cross-section (FEM–Beam model elliptical (rigid nodes), blue cross), and a model characterized by rigid nodes and circular strut cross-section (FEM–Beam model Gauss (rigid nodes), purple squares). The cross-section diameter is derived from the optical measurements of the struts (the Gauss best fit function; Section 2.4).

Different trends occur when nonlinear material behavior is considered. Plastic deformation influences the calculated compressive modulus of the structure in the linear regime. Despite this fact, the calculated results of compressive modulus are still in good agreement with the experiment (see Figure 17a FEM–Beam model). Even more accurate results are achieved if rigid nodes in the vicinity of the lattice structure strut nodes are considered (FEM–Beam model (rigid nodes)). Contrary to this, calculations considering elliptical (FEM–Beam model ellipse (rigid nodes)) or Gauss circular (FEM–Beam model Gauss (rigid nodes)) cross-section exhibit higher percentage values of structure compressive modulus *E_c_* compared to experiments. The biggest difference is visible when the values of structures with strut diameters 0.6 mm and 0.7 mm are compared.

The different trend of deviations occurs when the elastic-plastic material behavior is considered beyond the linear deformation of the structure. The deviation of engineering stress-strain dependency in this area indicates the beginning of structure collapse–Zone of active energy absorption. The first calculations performed with the model considering the nominal strut diameter (see Figure 17b FEM–Beam model) showed overall lower values of engineering stress with 0.2% deformation beyond the linear regime (collapse strain). The decrease is more significant with a rising strut diameter up to 0.9 mm, which corresponds to only 60% of the experimental stress. Contrary to this, slightly more accurate results achieve computations considering rigid nodes and circular Gauss cross-section (FEM—Beam model Gauss (rigid nodes)). Up to a diameter equivalent to 0.5 mm, the computation achieved engineering stress higher than experimental. Then, it started to decrease with a rising strut diameter up to the diameter equivalent to 1.0 mm, which corresponds to 70% of the experimental stress. Even closer to the experiment result an analysis can be achieved considering rigid nodes and elliptical cross-section (FEM–Beam model ellipse (rigid nodes)), which achieves the lowest value of 81% experimentally measured stress for the strut diameter equivalent to 1.0 mm.

A similar trend can be observed for the considered strut diameters also when the engineering stress (plateau stress) at 30% deformation is compared (see Figure 17c). According to the expectations, the lowest engineering stress achieves the calculation considering the nominal strut diameter without increased stiffness in the near vicinity of structure nodes (FEM–Beam model). Overall, lower engineering stress also leads to lower absorbed energy during the lattice structure deformation (see Figure 17d). Supplementing the model with rigid nodes and modified cross-sections leads to a more accurate prediction of the collapse stress in the FE analyses (see Figure 17b, FEM–Beam model ellipse (rigid nodes), and FEM–Beam model Gauss (rigid nodes)). The stress values of the performed analysis that consider the elliptical strut cross-section closely approach the experimental results (up to the nominal diameter of 0.7 mm). Beyond the linear deformation of the structure, a small influence of rigid nodes was observed, and therefore, its separate meaning is no further described in graphs.

The overall levels of engineering stress compared to the experiments in the area of lattice structure progressive collapse exhibit lower values with increasing nominal strut diameter. This behavior could be caused by the same issue that occurs when the material properties of lattice structures are determined based on conventional bulk samples (see Section 2.3) [23,31]. Furthermore, the internal area of tensile sample struts is usually manufactured with process parameters and strategies that differ from those that are applied to surface and subsurface areas because of the manufacturing technology. It leads to different values of mechanical properties. As the strut diameter changes, the ratio of both types of areas changes, and the mechanical properties are expected to variate. Therefore, the material properties should be determined for every strut configuration separately.

On the opposite, the tangent modulus values *E_ts_* (see Figure 17e) seem to be in a good correlation with the experiment for nominal strut diameters up to 0.8 mm diameter (FEM—Beam model). Above this strut diameter only models supplemented with geometrical imperfections can provide reasonably good results.

According to the FE simulations, an increment of partly melted material has a bigger significance on the loading force transmission. The deviations of the actual strut diameter can be caused by the different heat conductivity of powder and base parts, which causes the melting of the material with different intensities. This finding has a limitation because only a small range of structure geometrical configurations was tested (nominal CAD diameter ≤ 1 mm).

#### 3.4.3. Comparison with Specific Structured Component

**Figure 18:**
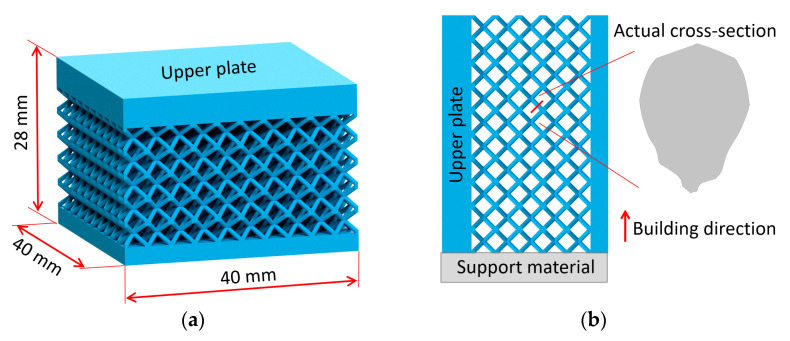
(**a**) Geometry of verification part; (**b**) building configuration with oriented cross-section imperfections.

**Figure 19:**
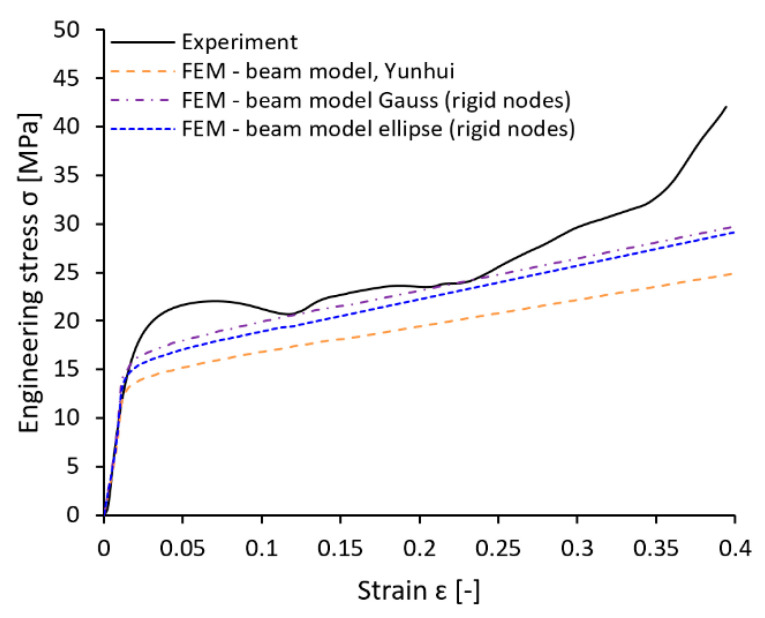
Engineering stress-strain comparison of reference structured part.

A further comparison of experiment and FE simulation was performed to verify the computational approaches in terms of material and geometry. A BCC lattice structure sample with dimensions of 40 × 40 × 20 mm^3^ (see Figure 18a) was manufactured with a bottom and upper plate with a thickness of 3 mm and 5 mm. A nominal strut diameter of 0.8 mm was chosen for the structure. The size of the unit cell remained the same as in the previous series. The experiment of quasi-static compression was performed under the same conditions described in Section 2.5. The resulting engineering stress-strain curve was compared to the simulations using the beam element models introduced in Section 3.4.2. (see Figure 19). The nonlinear behavior of the material was considered.

The verification part had to be manufactured rotated about 90° (see Figure 18b) due to the technological limits of the SLM process. Therefore, the geometrical imperfections that occurred in the manufacturing process were also oriented differently. This orientation was reflected in the model of geometry in simulations considering the elliptical cross-section of the strut.

The results showed a different level of engineering stress deviation for each computational model and experiment. In the first stage of structure loading, the compression modulus determined by simulation and experiment exhibits similar behavior. The difference occurs when the collapse strain is reached. According to the expectations, the worst result achieves for the model that considered the nominal strut diameter (FEM—Beam model, see Table 4). The model reaches only 75% of the experimentally determined stress at 0.3 strain. Compared to that, models supplemented with geometrical imperfections achieve far better results. The simulation considering the elliptical cross-section (FEM—Beam model ellipse (rigid nodes)) reaches 87% of the experimentally determined stress at 0.3 strain, and the simulation considering Gauss circular cross-section (FEM—Beam model Gauss (rigid nodes)) reaches even 89%. Similar results can be achieved when the stress level at 10% and 20% of deformation or absorbed energy is compared (see Table 4).

In contrast to the previous comparison in Figure 17c, it seems to be more efficient to use a Gauss circular cross-section instead of an elliptical for a nominal strut diameter of 0.8 mm in simulations. However, it must be mentioned that the results obtained by simulations of samples with different strut diameters focused mainly on the description of geometrical imperfections in the loading direction, which corresponds to the building direction (where imperfections manifest probably the most, see Figure 7). A different situation can occur when other loading directions are considered. Therefore, imperfections in directions that do not correspond to the building direction could be better described by different cross-section approximations. To confirm this hypothesis, a further comparison of imperfections’ influence on the mechanical properties in different loading directions has to be done.

## 4. Conclusions

The quasi-static compression behavior of the BCC lattice structure made of stainless steel 316L by selective laser melting technology was investigated experimentally, analytically, and through finite element modeling. A good correlation between the experiment and analytical-based approach using the Timoshenko theory was achieved for the equivalent of elliptical cross-section (up to 12% within diameter 0.5 mm). Analytical approaches were further supplemented with numerical simulations. In the first step, a nominal CAD-designed geometry discretized by Timoshenko beam elements and solid tetrahedron elements was used. A linear elastic material behavior was used for the simulation. In the second step, two additional numerical approaches considering geometrical imperfections were introduced into the simulation with the non-linear elastic-plastic model of the material. The main conclusions of this study can be described in the following points:

1. It is efficient to use specially designed tensile samples that consist of more thin struts to determine the actual mechanical properties of lattice structures. A good correlation (up to 5%) between mechanical properties determined in this study and described in the literature [32] was found. The analytical models support the credibility of the mechanical properties in the linear-elastic regime;
2. The geometrical imperfections can acquire different significance across variating strut diameter for one structure manufactured with the same process parameters and different geometrical parameters, e.g., strut diameter;
3. The FE analyses with solid and beam element models can predict the lattice structure compressive modulus with similar accuracy if an artificial stiffness increase in the vicinity of nodes is used within the beam element model;
4. The significance of geometrical imperfections increased after reaching 0.2% deformation beyond the linear regime (collapse strain). Including the imperfections improve the accuracy of calculations for both introduced approaches, whereas the change of cross-section to the elliptical seems to be more effective than the change to Gaussian circular for all diameters in the tested range;
5. The calculated levels of engineering stress compared to experiments in the area of lattice structure progressive collapse (30% deflection of structure) exhibit lower values with increasing nominal strut diameter. This phenomenon can indicate different values of mechanical properties of different strut diameters;
6. According to the FE simulation, an increment of partly melted material has a bigger significance for the loading force transmission. The finding is similar to the study of Vrana [31], who determined geometrical imperfections for AlSi_10_Mg with similar methods. It would be interesting to investigate the strut diameters beyond the range of diameters in this study (nominal CAD diameter > 1 mm) to determine the influence of the described imperfections in the future.

## Figures and Tables

**Table 1:** Chemical analysis of TLS stainless steel 316L powder.

Elem.	Fe	C	Si	Mn	Cr	Mo	Ni
wt.%	Bal.	0.03	0.8	1.8	17.5	2.2	11.3

**Table 2:** Average strut dimensions according to different measurement methods.

d_n_	d_Gauss_	S_Gauss_/S_n_	d_maj_	d_min_	S_ellipse_/S_n_
(mm)	(mm)	(%)	(mm)	(mm)	(%)
0.3	0.34	+13.7	0.39	0.27	+41.4
0.4	0.49	+21.5	0.60	0.35	+108.3
0.5	0.59	+18.0	0.73	0.44	+46.2
0.6	0.68	+13.4	0.81	0.55	+34.9
0.7	0.77	+10.5	0.93	0.61	+33.2
0.8	0.86	+7.9	0.99	0.72	+23.8
0.9	0.94	+4.1	1.06	0.82	+18.1
1	1.04	+4.2	1.15	0.92	+14.9

**Table 3:** Comparison of mechanical properties of conventional samples and samples with thin struts.

	*E_s_*	*R_p_*_0.2%_	*E_t_*	*R_m_*	*A*
	(GPa)	(MPa)	(MPa)	(MPa)	(%)
Multi-strut tensile samples	94 ± 10	338 ± 20	787	397	5.3
Conventional samples	166 ± 15	450 ± 5	89	541	40.7

**Table 4:** Comparison of stress and absorbed energy for different structure deflection.

	Experiment		Simulations					
			Beam Element		Beam Element Gauss		Beam Element Ellipse	
*Ɛ*	*σ*	*E_a_*	*σ*	*E_a_*	*σ*	*E_a_*	*σ*	*E_a_*
(-)	(MPa)	(MJ·m^−3^)	(MPa)	(MJ·m^−3^)	(MPa)	(MJ·m^−3^)	(MPa)	(MJ·m^−3^)
0.1	21.24	1.88	16.84	1.4	19.96	1.65	18.94	1.56
0.2	23.51	4.17	19.41	3.18	23.09	3.77	22.2	3.58
0.3	29.61	6.69	22.15	5.26	26.41	6.24	25.66	5.98

## Data Availability

The data presented in this study are available on request from the corresponding author.

## Abbreviations

AM	additive manufacturing
BCC	Body-centered cubic
FEA	finite element analysis
SLM	Selective laser melting
FCC	face-centered cubic
CAD	computer-aided design
µ-CT	micro computed tomography
*Q*_10_	10% quantile of particles distribution
*Q*_50_	50% quantile of particles distribution
*Q*_90_	90% quantile of particles distribution
$E_{c1}^{e}$	elastic modulus of lattice structure
$E_{s}$	elastic modulus of bulk material
$\upsilon_{s}$	Poisson’s constant of bulk material
*r_n_*	nominal strut radius
*l*	half of unit cell diagonal
*m*	weight of sample
*m_a_*	measured weight of sample
*m_n_*	nominal CAD weight of sample
*σ*_0.2%_	collapse stress (0.2% structure strain)
*R_m_*	ultimate tensile strength
*Φ_n_*	nominal volume fraction
*d_n_*	nominal strut diameter
*S_n_*	cross-section area of nominal strut diameter
*d_Gauss_*	diameter given by Gauss distribution
*S_Gauss_*	cross-section area of Gauss strut diameter
*d_maj_*	major axis diameter
*d_min_*	minor axis diameter
*S_ellipse_*	cross-section area of elliptical strut
*R_p0.2%_*	yield strength (0.2% proof stress)
*E_t_*	tangent modulus
*E_c_*	structure compressive modulus
*σ*	engineering stress
*Ɛ*	engineering strain
*E_ts_*	tangent modulus of structure
*A*	elongation at break
*E_a_*	volume energy absorbed
